# Functional Magnetic Resonance Imaging and Functional Near-Infrared Spectroscopy: Insights from Combined Recording Studies

**DOI:** 10.3389/fnhum.2017.00419

**Published:** 2017-08-18

**Authors:** Vanessa Scarapicchia, Cassandra Brown, Chantel Mayo, Jodie R. Gawryluk

**Affiliations:** Department of Psychology, University of Victoria Victoria, BC, Canada

**Keywords:** fMRI, fNIRS, multimodal, combined recording, cognition

## Abstract

Although blood oxygen level dependent (BOLD) functional magnetic resonance imaging (fMRI) is a widely available, non-invasive technique that offers excellent spatial resolution, it remains limited by practical constraints imposed by the scanner environment. More recently, functional near infrared spectroscopy (fNIRS) has emerged as an alternative hemodynamic-based approach that possesses a number of strengths where fMRI is limited, most notably in portability and higher tolerance for motion. To date, fNIRS has shown promise in its ability to shed light on the functioning of the human brain in populations and contexts previously inaccessible to fMRI. Notable contributions include infant neuroimaging studies and studies examining full-body behaviors, such as exercise. However, much like fMRI, fNIRS has technical constraints that have limited its application to clinical settings, including a lower spatial resolution and limited depth of recording. Thus, by combining fMRI and fNIRS in such a way that the two methods complement each other, a multimodal imaging approach may allow for more complex research paradigms than is feasible with either technique alone. In light of these issues, the purpose of the current review is to: (1) provide an overview of fMRI and fNIRS and their associated strengths and limitations; (2) review existing combined fMRI-fNIRS recording studies; and (3) discuss how their combined use in future research practices may aid in advancing modern investigations of human brain function.

## Introduction

Functional neuroimaging has enabled unprecedented investigation of the brain’s underlying cognitive processes. Today, the cornerstone of modern functional neuroimaging relies upon hemodynamic-based measures of brain function, the most prominent of which is blood oxygen level dependent (BOLD) functional magnetic resonance imaging (fMRI). Since the first studies using fMRI were published in the early 1990’s, it has become a mainstay in cognitive neuroscience research (Logothetis, 2008), with increasing interest in its clinical applications (Bauer et al., 2014; Lang et al., 2014). More recently, functional near infrared technology (fNIRS) has emerged as a lesser-known alternative to traditional hemodynamic-based approaches that promises to shed additional light on the functioning of the human brain in settings previously inaccessible to fMRI (Irani et al., 2007). The purpose of the current review is therefore to: (1) provide a brief overview of the basics of BOLD fMRI and fNIRS, including the strengths and limitations associated with these two techniques; (2) provide an overview of existing fMRI-fNIRS recording studies; and (3) discuss how their combined use in future research practices may aid in advancing modern investigations of human brain function.

## Overview of Functional Magnetic Resonance Imaging and Functional Near Infrared Spectroscopy

### Functional Magnetic Resonance Imaging

The physical principles of magnetic resonance imaging (MRI) are based on the theory of nuclear magnetic resonance (NMR) as pioneered by the early works of Edward Purcell and Felix Bloch (Bloch, 1946; Purcell et al., 1946). To very briefly summarize, according to NMR theory, all atomic nuclei, including hydrogen protons present in human tissue, possess an intrinsic magnetic moment or *spin*. When placed in a magnetic field, as occurs in an MRI scanner, the majority of hydrogen nuclei align parallel to the main magnetic field and rotate with a frequency that is proportional to the field strength. Exposure to radiofrequency pulses at the same frequency as the precessing hydrogen nuclei (which become unique as a result of overlaid magnetic field gradients) non-invasively displaces the nuclei, resulting in a net transverse magnetization vector (Weishaupt et al., 2008). The return of these nuclei to equilibrium creates a distinctive signal within different tissue types; the electromagnetic signal can be measured within each voxel, thereby generating the high-resolution images characteristic of MRI (van Geuns et al., 1999).

In addition to structural imaging of the brain, functional MRI has made it possible to examine active brain regions. Typically, fMRI relies on the BOLD contrast. The source of the BOLD contrast is derived from the difference in magnetic susceptibility of deoxygenated blood, which is paramagnetic (attracted to an external magnetic field) and oxygenated blood, which is diamagnetic (repelled from an applied magnetic field; Kim and Bandettini, 2012). This concept was first described in a series of seminal studies by Ogawa and colleagues (Ogawa and Lee, 1990; Ogawa et al., 1990a,b), who demonstrated that the presence of deoxyhemoglobin generates local magnetic field inhomogeneities in blood vessels and surrounding tissues in gradient-echo MRI. It is thought that these field inhomogeneities trigger a phase-dispersion of the proton signal, which in turn reduces the MR signal intensity (Ogawa et al., 1990a). Conversely, the presence of oxygenated hemoglobin (Hb) has been shown to lessen these paramagnetic effects, thereby leading to an *increase* in the BOLD signal intensity (Buxton, 2009). The result of this is therefore an MR contrast that varies according to the levels of blood oxygenation (Ogawa et al., 1990a). Notably, this change in oxygenation is itself determined by other variables such as the rate of oxygen consumption and regional cerebral blood flow (CBF) and volume (Ogawa et al., 1993).

Conventionally, it is thought that when a brain region becomes engaged in a task there is an associated increase in metabolic demands that must be supported. Consequently, regional increases in CBF and volume occur to deliver oxygenated blood to active neurons. As required, deoxygenated blood is produced, although a surplus of oxygenated blood remains, resulting in a small measurable increase in signal intensity (Roy and Sherrington, 1890). Thus, the overarching theory is that the BOLD signal serves as a proxy measure of the neural activation elicited by a stimulus. This basic principle has been supported by a number of combined fMRI and electrophysiological recordings which have found a linear relationship between spatial increases in the BOLD signal and increased regional neural activity (Logothetis et al., 2001). Notably, this increase in the BOLD signal has been shown to reflect the local field potentials caused by incoming neural input and intracortical processing, rather than action potentials (Logothetis et al., 2001; Logothetis, 2002).

#### Strengths and Limitations

Many of the reasons for which fMRI BOLD has emerged as a method of choice are straightforward: it is non-invasive, repeatable, widely available and has superior spatial resolution compared to other neuroimaging techniques (Logothetis, 2008). Furthermore, the high-resolution activation maps produced by fMRI allow for visually communicable results, which makes this technique more accessible to non-expert audiences. However, fMRI, like all neuroimaging technologies, also has a number of limitations. Most notably, there are several practical constraints that limit the ecological validity of the tasks that can be performed in an MRI scanner, namely: contraindications to being in a magnetic field (e.g., metal in the body from a previous surgery), claustrophobia, restrictions on movement, the typical supine position of subjects, the requirement for compatible response equipment, and the noise produced by the scanner (Logothetis, 2008). Functional MRI studies can also be expensive, given the high costs required to maintain the equipment and employ technicians to operate the scanner.

Furthermore, the relationship between neural activity and the BOLD contrast is one that is highly complex: signal increases in fMRI are inherently linked to a number of physiological variables, including the efficiency of the hemodynamic response and the unique properties of the neural circuit being interrogated (e.g., cell type, inhibitory/excitatory synaptic activity; Logothetis and Wandell, 2004). As argued by Logothetis and Wandell (2004), these factors inherently limit the interpretation and “visibility” of brain function that is possible with BOLD fMRI alone. Indeed, relevant to the current review, these limitations have necessitated the use of optical imaging technology to shed further light on the spatiotemporal elements that underlie the BOLD signal (Uludağ, 2010).

In spite of these practical limitations, considerable advances in understanding the neural activity underlying cognitive functions have been made possible with fMRI and it is currently considered the gold standard non-invasive hemodynamic based neuroimaging technology. Indeed, functional MRI has greatly contributed to our current understanding of the functional neuroanatomy of cognition in both healthy younger (Cabeza and Nyberg, 2000; Cabeza, 2001) and older adults (Cabeza, 2001). However, imaging technology is increasingly being applied to examine brain activity during dynamic behaviors such as exercise (e.g., Fontes et al., 2015) and being used in populations that deviate from typical age and cognitive norms (e.g., infants, abnormal aging; Fransson et al., 2009; Sperling, 2011). This increases the need for techniques that enable a precise understanding of brain function that is both ecologically valid and amenable to populations and contexts that may not be well suited to standard fMRI protocols. While ingenuity and well-executed experimental designs can still make fMRI valuable in beginning to address such issues, there is room for additional approaches, particularly those with complementary strengths where fMRI is limited. To this end, it has been suggested that multimodal imaging will be essential in making further progress in the understanding of brain-behavior relationships (Muthalib et al., 2013).

### Functional Near-Infrared Spectroscopy

One technology that shows increasing promise as an independent functional neuroimaging method and for use in conjunction with MRI and fMRI is known as fNIRS. The development of fNIRS emerged from Jöbsis’ (1977) reporting that near-infrared light can be effectively transmitted through biological tissues over large distances. This observation enabled the first *in vivo* detection by this group of cortical oxygenation in humans during anoxic conditions (Jöbsis, 1977). Despite these earlier efforts, its uptake as a research and clinical tool has been considerably slower, with the emergence of the first cluster of studies demonstrating its utility as a neuroimaging technology in 1993 (Chance et al., 1993; Hoshi and Tamura, 1993a,b; Okada et al., 1993; Villringer et al., 1993). Commercial clinical instruments were first available in 1989 but initial instruments were quite limited (Ferrari and Quaresima, 2012). More recently, fNIRS systems with multiple probes and more advanced analysis packages have enabled broader use in research and clinical practice (e.g., Lv et al., 2008; Huppert et al., 2009).

Modern fNIRS is a non-invasive optical imaging technique that can be used to measure changes in Hb concentrations in brain tissue (Hoshi, 2005; Huppert et al., 2009; Ferrari and Quaresima, 2012). Like fMRI, fNIRS measures the hemodynamic response to neural activity, but rather than relying on the paramagnetic properties of Hb, it relies on the different absorption properties of biological chromophores (Hoshi, 2005; Ferrari and Quaresima, 2012). A biological chromophore is a group of atoms within a molecule that generates its color through the absorption of light at different wavelengths; this absorption may also include light in the non-visible spectrum, such as longer (infrared) or shorter (ultraviolet) wavelengths (Lackie, 2010; Colman, 2015).

As per Jöbsis’ (1977) initial reports, near-infrared light, between 700 nm and 900 nm, easily passes through biological tissue because the light of these wavelengths is only absorbed by a few biological chromophores: Hb, myoglobin (Mb) and cytochrome oxidase (CytOx) in the mitochondria (Ferrari and Quaresima, 2012). Because the spectra of Hb and Mb vary in their oxygenation state, information about their oxygenation-deoxygenation states can be obtained from the NIR light transmitted through the tissue resulting in measures of oxygenated Hb (oxy-Hb) and deoxygenated Hb (deoxy-Hb; Hoshi, 2005). The spectrum of CytOx varies with oxidative state, allowing this to also be detected with NIR systems; however, this latter measure is uninteresting from the perspective of functional neuroimaging because changes only occur under severely hypoxic conditions (Hoshi, 2005; Ferrari and Quaresima, 2012).

Functional NIRS research is rapidly expanding across a wide range of areas (Boas et al., 2014). Active areas of research using fNIRS include neurodevelopment in infants (Boas et al., 2014), and the neural correlates of cognitive functions including verbal fluency (Schecklmann et al., 2008), language (Quaresima et al., 2012) and working memory (Kwee and Nakada, 2003). Functional NIRS has also been used to investigate the hemodynamic response during activities that cannot be measured within a fMRI scanner such as walking (Suzuki et al., 2008; Holtzer et al., 2011), exercise (Yanagisawa et al., 2010), imitation of another person (Moriai-Izawa et al., 2012) and brain activity during everyday tasks such as apple-peeling (Okamoto et al., 2004b).

#### Strengths and Limitations

Functional NIRS has a number of advantages that make it an ideal choice for interrogating brain function in contexts that may be inaccessible to fMRI. To begin, in terms of its utility as a research tool, fNIRS is non-invasive, relatively inexpensive and has a temporal resolution that is comparable to that of fMRI (Hoshi, 2005; Quaresima and Ferrari, 2016). However, perhaps the most characteristic feature of fNIRS is its portability: recent technological advancements in modern fNIRS systems have resulted in devices that are increasingly miniaturized, wireless and battery-operated (Ayaz et al., 2013; McKendrick et al., 2016). This has allowed for advancements in the study of neurocognitive processes in unconstrained environments, including studies outdoors and in various other ambulatory settings (Balardin et al., 2017; McKendrick et al., 2017). To this end, one of the most notable advantages of fNIRS as a neuroimaging tool is the lack of strict restrictions on motion. This stands in stark contrast to fMRI, where complete stillness is required during the acquisition in order to ensure proper data quality and the absence of motion artifacts.

Though significantly more tolerant of head-motion than fMRI, fNIRS signals are still sensitive to degradation due to motion artifacts. However, several groups have elaborated on methods for real-time motion correction that allow for exploitation of this method’s greater portability (Cui et al., 2010; Izzetoglu et al., 2010; Falk et al., 2011; Strait and Scheutz, 2014). This makes fNIRS an attractive alternative, particularly for populations where complete stillness can be extremely challenging, such as infants. Moreover, portable fNIRS devices also allow for paradigms that would be impossible with fMRI, such as investigating the neural correlates of walking (Perrey, 2014; Piper et al., 2014), and can also be employed to more closely parallel the conditions under which a clinical neuropsychological assessment would actually take place (e.g., face to face with an examiner, Moriai-Izawa et al., 2012). Furthermore, as functional neuroimaging becomes an increasingly important component of clinical research, the insensitivity of fNIRS to common electrical or magnetic devices, such as hearing aids, pacemakers, or cochlear implants, also serves as a significant advantage where fMRI is limited (Quaresima and Ferrari, 2016).

Finally, though beyond the scope of the current review, another strength of fNIRS is the ease with which it may be integrated with other neurocognitive applications. In addition to fMRI, recent technological advances have centered on the combined technological and functional uses of fNIRS and electroencephalography (EEG), with the goal of advancing brain-computer interface technologies (e.g., Sood et al., 2016; von Lühmann et al., 2017). Moreover, beyond merely imaging modalities, fNIRS may also be used to augment and further elucidate the mechanisms underlying neurostimulation techniques, including transcranial direct current stimulation (McKendrick et al., 2015).

The main limitations of fNIRS as a clinical neuroimaging tool relate to physical and technological constraints imposed by the device setup. Modern fNIRS instruments consist of source and detector probes positioned on the scalp typically at a fixed distance apart, ranging from 2 cm to 5 cm (Hoshi, 2005; Ferrari and Quaresima, 2012). The most commonly used commercially available fNIRS systems are referred to as continuous wave instruments, so-named for their continuous emission of light at a constant amplitude. From this, changes in chromophore concentrations can be calculated according to the modified Beer-Lambert law (Delpy et al., 1988). Continuous wave-type instruments do not provide absolute values of concentration changes because the length that light travels through the tissue is unknown. However, relative concentration changes can be calculated by substituting an arbitrary unit, while path length can be calculated through co-registration with anatomical images from MRI (Hoshi, 2005; Huppert et al., 2009; Ferrari and Quaresima, 2012).

Functional NIRS is also limited in the information that it can provide about cortical activity. Unlike fMRI, which is capable of whole brain measurement, the number of sources and detectors in the fNIRS setup determines the size of the brain area that can be measured, which is often limited to frontal regions. Similar restrictions on measurement also apply to brain depth: while fMRI is capable of detecting activity in deep cortical and subcortical regions, fNIRS is only able to detect NIR light that penetrates the first few centimeters of cortical tissue, making this one of fNIRS’ key limitations (Hoshi, 2005; Ferrari and Quaresima, 2012). Moreover, as with fMRI, fNIRS signals are contaminated by physiology-based interference from cardiac pulsation, respiration and a variety of spontaneous low frequency oscillations occurring in the range of 0.01–0.1 Hz (Obrig et al., 2000). These low frequency oscillations include spontaneous changes in local vascular tone and more systematic changes in blood pressure (Obrig et al., 2000). Similar to fMRI data processing, a variety of processing methods have been developed to remove these frequencies from the data, the most common of which are *low-pass filters* to remove high-frequency instrument noise and fast cardiac oscillations, and *high-pass filters* to remove low-frequency physiological noise (Huppert et al., 2009). Unlike fMRI, however, fNIRS signals also contain superficial scalp signals embedded in the, usually more desired, “brain signal” (Erdoğan et al., 2014). Moreover, activation-related fNIRS signals can also arise from all vascular compartments, including arterioles, venules and capillaries, which can render more complex the precise interpretation of fNIRS signals (Liu et al., 1995). Finally, functional NIRS also lacks anatomical information and thus relies on co-registration with either structural MRI from each participant or the 10–20 system (Okamoto et al., 2004a,b).

For an overview of the strengths and limitations of fMRI and fNIRS, see Table 1.

**Table 1:** An overview of strengths and limitations associated with fMRI and fNIRS.

	fMRI	fNIRS
**Strengths**	Non-invasive Repeatable Widely available Superior spatial resolution Whole brain measurement (lateral surface and depth)	Non-invasive Repeatable Comparable temporal resolution to fMRI Inexpensive Portable Less restriction on motion
**Limitations**	Expensive Strict restrictions of motion Need for supine position Noisy scanner Physiological noise Restrictions based on metal in the body Restrictions based on claustrophobia	Limited to frontal regions and surface analysis Physiological noise (including superficial scalp signals) Lacks anatomical information Interpretation challenges related to multiple sources of vascular signal

## An Overview of Combined fMRI-fNIRS Studies

A survey of the literature in this area to date yields just over 100 published articles using combined fMRI-fNIRS investigation in studies of brain function. Given the relatively novel emergence of fNIRS as an alternative hemodynamic-based imaging tool, a primary focus of many of these studies is that of validating fNIRS hemodynamic measures against the extensively studied gold standard fMRI BOLD signal. However, functional MRI-fNIRS studies have also been used to investigate the BOLD signal itself: while fMRI is sensitive to the paramagnetic properties of deoxy-Hb—such that there is an increase in signal with increased oxygenated blood—fNIRS provides the added advantage of separately measuring deoxy-Hb, oxy-Hb and total hemoglobin (total-Hb) and their relative contributions to measures of “activation”. Thus, where the unique specifications of both fMRI and fNIRS are combined in such a way that the two procedures complement each other, multimodal imaging studies may assist in narrowing the gap between research and practice by allowing for more complex research questions and ecologically valid experimental designs than is feasible with fMRI alone (Noah et al., 2015).

One important consideration in fMRI-fNIRS studies is the lack of certainty around which fNIRS parameter, or combination thereof, should best correlate with the fMRI BOLD signal. In a review of combined fMRI-fNIRS studies, Steinbrink et al. (2006) concluded that the temporal correlation of the BOLD signal and fNIRS measured changes in deoxy-Hb was generally considered the common denominator of the two methods. However, some studies have also demonstrated a better temporal correlation with total-Hb (Hess et al., 2000; Strangman et al., 2002). Given that both deoxy-Hb and cerebral blood volume contribute to the BOLD signal, it has been suggested that the fNIRS parameter that best correlates with the BOLD signal may depend on physiological and measurement noise (Cui et al., 2011).

The potential utility of fNIRS as a cheaper and more naturalistic method of investigating expected activation based on previous fMRI findings (Kwee and Nakada, 2003) have also led to a number of studies examining the source of brain “activation” as identified by fNIRS vs. fMRI. To date, investigations comparing fNIRS and fMRI have been conducted for a variety of tasks, including motor (Cui et al., 2011; Noah et al., 2015; Anwar et al., 2016), visual (Sakatani et al., 2007; Fabiani et al., 2014) and language tasks (Quaresima et al., 2012; Vannasing et al., 2016), as well as tasks with expected frontal activations, such as working memory (Sato et al., 2013; Ozawa et al., 2014). Finally, some groups have also begun to examine the relationship between fMRI and fNIRS in the context of resting state networks (Duan et al., 2012; Sasai et al., 2012).

### Motor-Sensory Tasks

In a comprehensive multimodal imaging study, Cui et al. (2011) scanned participants with fMRI, while the right frontal cortex and right parietal cortex signals were measured with fNIRS. Participants completed four tasks: left finger tapping, a go/no-go task, judgment of line orientation and N-back. Regions of interest (ROIs) were defined as the ellipsoid shape between the light emitting fNIRS probe and the detector. Correlations were calculated between the fNIRS total-Hb signal and the fMRI BOLD signal measured at each voxel within the predicted ellipsoid. The results of this study found that correlations between the fMRI and fNIRS time courses (deoxy-Hb, oxy-Hb and total-Hb) in the corresponding ROI ranged from as high as 0.8 to as low as 0, with the mean remaining significantly different than 0. Overall, oxy-Hb had the strongest correlation with fMRI BOLD. Although there were no significant differences by task type, correlations did vary by region, with higher correlations identified in middle frontal and inferior parietal regions relative to other areas of the brain. It was also found that the scalp-brain distance, which also varies according to region, was *negatively* related to the fNIRS-fMRI correlation, that which is thought to be due to a degradation of fNIRS signal quality with increasing distance. Based on these findings, the authors suggest that frontal regions might have a double advantage as areas of investigation for fNIRS, in that they are both shorter in scalp-brain distance and hairless (which reduces scattering and facilitates probe placement). On the basis of their findings, the authors concluded that motor task activation patterns in the motor cortex are similar for both modalities and across cognitive tasks, but inferior in resolution when assessed with fNIRS (Cui et al., 2011).

In another multimodal study of basic motor activation, Anwar et al. (2016) conducted a simultaneous fMRI-fNIRS-EEG experiment to examine cortico-cortical sensorimotor networks during a variety of right hand finger movements, ranging from single finger tapping to more complex finger sequences. The *effective connectivity* between three cortical ROIs (the contralateral sensorimotor cortex, the contralateral premotor cortex and the contralateral dorsolateral prefrontal cortex) was investigated using what is known as the Granger causality (CG) analysis. Their results revealed a bi-directional flow of information between all three ROIs, as determined by both hemodynamic measures (fMRI and fNIRS) as well as EEG. While this effect was not found to vary according to the complexity of the task, their findings did reveal that EEG provided the largest CG amplitudes relative to the hemodynamic measures, both of which performed equivalently. The authors suggest that, while EEG may be the best technique to examine the directionality of neural communication in the brain, both fMRI and fNIRS may be used to guide source localization of sensorimotor networks, with fNIRS serving as an attractive alternative to fMRI for bedside multimodal investigations that wish to combine the temporal sensitivity of EEG with the spatial resolution of hemodynamic measures (Anwar et al., 2016).

Even for basic motor-sensory tasks, one of the challenges in comparing fMRI and fNIRS measurements lies in the inherent difficulty of reconstructing images of brain activation as detected by fNIRS (Huppert et al., 2017). As such, many investigations have been limited to patterns of temporal correlation. In light of this, Huppert et al. (2017) sought to perform a *quantitative* comparison of fMRI-fNIRS spatial activation patterns using a novel image reconstruction method by this same group that is based on cortical surface topology (Abdelnour et al., 2009, 2010; Huppert et al., 2009; Abdelnour and Huppert, 2011; Barker et al., 2013). Specifically, they examined concurrent fNIRS-magnetoencephalography (MEG) and fNIRS-fMRI during a somatosensory stimulation paradigm consisting of variable patterns of right median nerve stimulation. Their results revealed close agreement between all three modalities, with spatial correlations of *r* = 0.57 and −0.48 for fMRI-fNIRS oxy-Hb and fMRI-fNIRS deoxy-Hb, respectively. Importantly, the majority of the observed differences between fMRI, fNIRS and MEG were found to be driven by the lower sensitivity to subcortical signals in the MEG and fNIRS modalities (Huppert et al., 2017), again supporting the potential benefits of a complimentary, multimodal approach.

While studies such as those by Cui et al. (2011), Anwar et al. (2016) and Huppert et al. (2017) have provided compelling evidence for the ability of fNIRS to replicate brain activation patterns recorded with fMRI during simple motor-sensory tasks such as finger movements, far fewer studies have examined the potential of multimodal fMRI-fNIRS imaging as a tool for studying full-body behaviors. In light of this gap in the literature, a recent study by Noah et al. (2015) sought to examine the correlation between brain activity recorded with fNIRS during a dance video game task, to activity patterns captured by fMRI during a scanner-compatible version of the task. The ROI was defined as the superior and middle temporal gyrus, based on a previous fNIRS study by this group that examined a similar interactive dance task (Ono et al., 2014), and the known involvement of this region in motor-sensory integration. Their results revealed a high correlation (*r* = 0.78) between fNIRS signals in the ROI during the naturalistic task and fMRI activity captured during the reduced version of the task. Though the high correlation between the two signals provides promise for the utility of fNIRS in examining tasks previously inaccessible to traditional neuroimaging techniques, the authors were careful to note that fNIRS will not replace the need for fMRI. Rather, they argue that data obtained from the two imaging techniques may be used to complement one another in the case of individuals with conditions that render fMRI less feasible or valid, such as those with tremor, dyskinesia, or implanted devices—all of whom may benefit from advancements in imaging-based neurorehabilitaion (Noah et al., 2015).

### Cognitive Tasks

Sato et al. (2013) conducted a multimodal study employing fNIRS, fMRI and laser Doppler imaging to examine prefrontal cortex activity during working memory and motor tasks. The results of this study revealed strong correlations between the fMRI BOLD time course and fNIRS measures of the hemodynamic response: in a block averaged comparison, task-related signal changes in fMRI BOLD and fNIRS oxy-Hb showed a positive correlation, while deoxy-Hb was negatively correlated with the BOLD signal. Notably, this study also identified a correlation between the *amplitudes* of the fMRI BOLD signal and both fNIRS oxy-Hb (*r* = 0.65) and deoxy-Hb (*r* = −0.76) in the prefrontal cortex during the working memory task. Moreover, an important secondary finding from this study was that, across different tasks with frontal lobe activations, fNIRS hemodynamic measures tended to correlate better with BOLD signal located in the gray matter than with superficial Doppler skin blood flow measures in the areas of activation (Sato et al., 2013). This stands in contrast to an earlier study, which raised concerns about the source of fNIRS measured changes in oxy-Hb when they found that frontal activations disappeared when participants restricted blood flow to the region with a finger press during verbal fluency test performance (Takahashi et al., 2011).

In another simultaneous recording study of a frontally-mediated, intertemporal choice of reward task, fMRI and fNIRS also showed similar areas of activation, namely a cluster of activation in the right middle and inferior frontal gyrus, but lower mean temporal correlations (mean |r| ~ 0.2; Heinzel et al., 2013).

Further evidence for the compatibility of fMRI and fNIRS in neurocognitive investigations comes from multimodal studies that have incorporated advances in imaging technology to transform channel-based fNIRS signals to voxel-based fNIRS “cortical activation maps”, akin to those used in fMRI (Wijeakumar et al., 2017). Using this methodology, Wijeakumar et al. (2017) examined the neural correlates of a visual working memory task and found significant *voxel-wise* correlations between fMRI and fNIRS in the frontal, parietal, temporal and occipital cortices. Specifically, fNIRS oxy-Hb showed a positive correlation with the BOLD signal for all experimental conditions, whereas deoxy-Hb showed a negative correlation with the BOLD signal. Moreover, this spatial overlap was maintained following a parametric modification of the task in which memory load was progressively increased, pointing to a highly robust and specific correlation of fMRI-fNIRS signal dynamics (Wijeakumar et al., 2017).

It is also worth noting that the combined uses of fMRI and fNIRS for studying human cognition are not solely limited to artificial tasks paradigms conducted in laboratory settings. Indeed, most recently, Liu et al. (2017) examined multiple brain monitoring during a naturalistic task of verbal communication. Specifically, they used fNIRS to examine the neural correlates of storytelling in one group of participants and audio comprehension of these same stories in a separate group of participants. They then compared the fNIRS-collected data to fMRI-acquired BOLD measures of listeners comprehending the same story while in the MRI scanner. In congruence with previous studies, the results of this investigation showed a significant correlation between fNIRS oxy-Hb and the fMRI BOLD signal in brain regions mediating language comprehension. Notably, these correlations remained despite the fNIRS and fMRI data being collected in different subjects and in different testing environments. Moreover, this correlation was found to disappear when different stories were compared between the two modalities, again pointing to a high level of specificity in the fMRI-fNIRS temporal association (Liu et al., 2017). Studies of this nature attest to the potential of advances in multimodal imaging to broaden the study of cognitive neuroscience to the socio-emotional realms of human cognition.

### Resting-State

Further evidence of the validity of fNIRS indices as measure of brain hemodynamic responses comes from fMRI-fNIRS investigations of resting state networks. In one of the first studies of this kind, Sasai et al. (2012) compared whole-brain resting state fMRI data to fNIRS signals simultaneously obtained in frontal, temporal and occipital regions. Interestingly, this study found that the fMRI voxels that best correlated with the resting-state fNIRS signal were those within two voxels from the estimated projection point of the fNIRS probe. Moreover, it was also found that oxy-Hb maps and fMRI BOLD maps had a significant positive correlation for all brain regions investigated, and that the correlation maps of three resting state networks (dorsal attention, fronto-parietal control and default mode) could be successfully reproduced using NIRS signals as seeds. On the basis of these findings, the authors conclude that fNIRS, like fMRI, may therefore serve as valid measure of resting-state cortical functional connectivity (Sasai et al., 2012).

Another simultaneous recording study by Duan et al. (2012) similarly demonstrated the ability of fNIRS to produce resting-state functional connectivity networks comparable to those produced by fMRI. Specifically, the results of this study found that fNIRS exhibited validity equivalent to fMRI in detecting the inter-individual strength of resting-state networks between participants. This was particularly true when the oxy-Hb signal was examined. Moreover, a linear regression analysis found that fNIRS could also account for a significant portion of the inter-regional variability in resting state networks strengths among individuals (*R*^2^ = 0.91 and 0.67, for oxy-Hb and deoxy-Hb, respectively), providing further support for the sensitivity of fNIRS as a neuroimaging technique (Duan et al., 2012). Thus, as a whole, fMRI-fNIRS studies to date have shown that fNIRS oxy-Hb and deoxy-Hb signals have the ability to reflect both hemodynamic changes due to spontaneous cortical activity and task-related cortical activation.

Other uses of combined fMRI-fNIRS resting-state studies have sought to further exploit the high sampling rate of fNIRS to examine physiological processes underlying the BOLD signal, such as physiological noise arising from cardiac pulsations, respirations, low-frequency oscillations, and other blood-related signal variations that are otherwise aliased by the low sampling rate of BOLD fMRI (Tong and Frederick, 2012). One central example of this is a study by Tong and Frederick (2012), which examined concurrent resting-state fMRI-fNIRS in healthy participants, wherein physiological signals acquired by fNIRS were used as regressors in the fMRI analysis to determine their impact on the BOLD signal using the Regressor Interpolation at Progressive Time Delays (RIPTiDe) method described previously by this group (Tong et al., 2011). Their findings revealed that cardiac signals most significantly affected voxels situated near large blood vessels, with low-frequency oscillations affecting regions proximal to drainage veins. On this basis, the authors suggest that the concurrent use of these imaging techniques may aid in advancing the study of cerebral hemodynamics in greater detail that has been previously possible with either technique alone (Tong and Frederick, 2012).

## The Merit of Combining fMRI and fNIRS: Avenues for Future Research

### Re-Examining the Hemodynamic Response

Simultaneous fMRI-fNIRS measurement can also provide insight into the fMRI BOLD signal due to the different indicators measured by fNIRS and fMRI (Steinbrink et al., 2006). While both fMRI and fNIRS are hemodynamic-based measures of brain activity that rely on the properties of neurovascular coupling, as mentioned previously, fMRI is limited in the information that it provides. For instance, while fMRI allows for a quantification of changes in signal intensity based on a combination of hemodynamic factors, including: (1) regional CBF; (2) cerebral blood volume; and (3) the cerebral metabolic rate of oxygen consumption (CMRO_2_), the majority of MRI acquisition techniques do not differentiate between these factors, resulting in a one-dimensional measure of brain signal intensity. As argued by Buxton et al. (2014), the popular notion that increased CBF necessarily results in a greater BOLD signal is one that is too simplistic and poses the risk of potentially clouding novel insights into neural activity in the human brain. As initially posited by Logothetis and Wandell (2004), the coupling of CBF and CMRO_2_ to neural activation in fMRI BOLD has been shown to vary according to variables such as the stimulus type and the particular brain region being interrogated (e.g., Chiarelli et al., 2007; Ances et al., 2008; Liang et al., 2013; for a review, see Buxton et al., 2014). As such, there is a greater need to interpret the BOLD signal in terms of its precise underlying physiological determinants (Buxton et al., 2014).

Steinbrink et al. (2006) reviewed joint fMRI-fNIRS studies in both human and animal subjects to illuminate the physiological principles and dynamics of the vascular response. Under conditions of sustained elevation of both perfusion and neuronal activity (as would be seen in typical young healthy populations) increased CBF results in a *decrease* in deoxy-Hb and *increase* in oxy-Hb. However, it is also posited that deviations to this general trend may occur in populations for whom CBF may be less responsive to challenge (Steinbrink et al., 2006; Obrig, 2014). This poses a significant challenge in studies of aging and cerebrovascular disease, where the interpretation of BOLD fMRI alone may be impacted by the above factors (D’Esposito et al., 2003). For instance, in a study of visual cortex activation in older adults, the fMRI BOLD response, acquired with an echo planar imaging sequence, showed no association with cardiovascular fitness (as measured by VO_2_ max). However, an association was observed with fNIRS: specifically, deoxy-Hb showed no significant effect of fitness, whereas the oxy-Hb response amplitude was correlated with VO_2_ max (Fabiani et al., 2014).

More recently, Alderliesten et al. (2014) conducted a study to compare parameters of oxygen metabolism acquired with BOLD fMRI and fNIRS during a respiratory challenge to better qualify the physiology underlying BOLD “activation”. Specifically, this group examined CBF and oxygen saturation from respiratory-calibrated fMRI and compared these changes to oxygen saturation, oxy-Hb, deoxy-Hb and total-Hb obtained with single-probe NIRS. Their results revealed that, out of all NIRS-derived parameters, total oxygen saturation showed the best correlation with the BOLD signal, with deoxy-Hb showing the best correlation with the BOLD signal out of all Hb measures. The authors suggest that knowledge of cerebral oxygen saturation, as estimated by fNIRS, may facilitate interpretation of the BOLD signal, and may furthermore widen the potential clinical applications of fMRI-validated fNIRS as a trend monitor of oxygen saturation in neonatal populations (Alderliesten et al., 2014).

Taken together, the ability of fNIRS to allow for quantitative monitoring total-, oxy- and deoxy-Hb in relation to a task, physiological state, or functional network provides highly germane information not otherwise accessible through existing neuroimaging techniques (Quaresima and Ferrari, 2016). This ability to refine our understanding of the relationship between neural activity and the BOLD signal is essential for accurate interpretation of fMRI BOLD activation—or lack thereof—particularly in patient populations where relations may be atypical due to changes in CBF and/or neurovascular structure (D’Esposito et al., 2003; Steinbrink et al., 2006; Sakatani et al., 2007; Obrig, 2014).

### Applications in Patient Populations

In other studies, fNIRS has also been used to specifically investigate variations in the BOLD response in patient groups. In particular, a study by Sakatani et al. (2007) exemplifies the aforementioned importance of using a multimodal approach in populations with altered neurovascular coupling. The study examined individuals with severe cerebral ischemia and found that BOLD fMRI showed weak activation on the lesion side in the primary sensorimotor area during a hand-grasping task, despite the participants’ successful completion of the task (Sakatani et al., 2007). However, in these same patients, fNIRS showed significant increases in deoxy-Hb, oxy-Hb, and total-Hb, thereby indicating that increased regional CBF did indeed occur (Murata et al., 2002; Sakatani et al., 2007). Notably, this pattern differed in control participants, who showed *decreased* deoxy-Hb and increased oxy-Hb and total-Hb in sensorimotor areas. It is therefore likely that disease-related abnormalities in the relative concentrations of oxy-, deoxy and total-Hb in the patient group resulted in a blunted fMRI BOLD response, as the latter technique is one that is primarily dependent on upon a relative surplus of diamagnetic oxy-Hb for reliable signal detection.

Another potential application of combined fMRI-fNIRS in patient groups includes optimization of neuroimaging techniques for the purposes of preoperative planning. For instance, a study by Fujiwara et al. (2004) used joint fMRI-fNIRS to examine primary sensorimotor cortex activation during hand grasping in a group of 12 participants with brain tumors (but without paralysis). The results of this study found that some of the participants exhibited decreases in deoxy-Hb on the ipsilesional side, while others actually exhibited increases deoxy-Hb on the ipsilesional side. Both groups, however, showed oxy-Hb and total-Hb increases during the task. While the latter fNIRS findings would indicate that regional CBF increases in response to neural activation occurred in both patient groups, fMRI BOLD alone revealed only small or no activation areas in the deoxy-increase group (Fujiwara et al., 2004).

To this end, in their brief review of combined fMRI-fNIRS investigations in stroke and brain tumor patients, Sakatani et al. (2007) conclude that using only fMRI BOLD preoperatively in brain tumor patients may result in the activation areas being identified as smaller than they really are. This poses significant barriers to the implementation of functional neuroimaging technology in clinical settings. As argued by Buxton et al. (2014), uncertainty surrounding the physiological underpinnings of the fMRI BOLD signal may also explain the lack of “clinical impact” of fMRI to date, despite extensive investigations and its clear latent potential to shed further light on disorders of the brain. This further highlights the need for multimodal imaging approaches in clinical research.

Finally, in addition to elucidating the physiological mechanisms underlying the BOLD fMRI signal in clinical populations, more recent research suggests that combined fMRI-fNIRS may also aid in improving the accuracy of BOLD fMRI signal detection in these groups. Indeed, it has long been known that physiological noise is a characteristic feature of the human nervous system (Stein et al., 2005; Faisal et al., 2008). While the source of this physiological noise remains unclear, as mentioned previously, is well established that variables such as heart rate, respiration and cardiac pulsatility exert a significant influence on fMRI measurements (Hu et al., 1995), often in the form of low-frequency oscillations in the data. As noted by Cooper et al. (2012), many fMRI experimental paradigms therefore rely on repeated task administrations in order to enhance the signal to noise ratio and overcome this important limitation of the hemodynamic response. The consequence of this is an increase in the scan duration, which can be especially problematic for studies in patient populations (Cooper et al., 2012). As described earlier in this review, previous studies suggest that a significant portion of the correlation between fNIRS and BOLD fMRI in the *resting-state* may actually be attributable to these low frequency oscillations arising from hemodynamic-based physiological factors (e.g., Tong and Frederick, 2010, 2012). Stemming from such findings, Cooper et al. (2012) sought to examine whether fNIRS signals may therefore be used as a *direct* regressor for physiological noise in fMRI data. The results of this study showed that, not only is fNIRS signal regression highly effective at reducing the hemodynamic error in resting-state BOLD fMRI, but it is also significantly *more* effective than other commonly used preprocessing approaches (e.g., RETROspective Image CORrection; RETROICOR). This alludes to an important role of multimodal imaging in the optimization of modern fMRI research in patient groups (Cooper et al., 2012).

### Considerations for Future Studies

The current review has centered on studies that have demonstrated both combined and concurrent uses of fMRI and fNIRS across both clinical and basic research domains. While the merit of multimodal imaging is clearly demonstrated in the discussions provided herein, the issue of simultaneous vs. combined acquisition is one that requires further consideration. For simultaneous acquisition in particular, a number of additional factors need to be considered, including the additional cost required to collect both modalities simultaneously and the relative merit gained over combined studies using advances in data fusion techniques (e.g., Yuan and Ye, 2013). Moreover, in addition to monetary costs, practical costs also need to be considered, including participant comfort and ease of setup. Collecting fMRI and fNIRS simultaneously may require additional time and effort to ensure that the fNIRS device is compatible with the magnetic resonance environment and is also able to accommodate a variety of head sizes in the limited space allotted in the scanner. For the reasons discussed above, combined applications of fMRI-fNIRS are likely sufficient for the majority of investigations looking to benefit from the advantages of multimodal imaging discussed in this review. One important exception may include studies that seek to more closely examine the mechanisms underlying the BOLD signal, in which case simultaneous acquisition may be necessary to more precisely map the fNIRS parameters corresponding to measures of fMRI “activation”. Careful consideration of the above factors are required on a study-by-study basis in order to benefit from the advantages of multimodal imaging, while also preserving the efficiency and feasibility of future investigations.

## Conclusion

The current review provided details regarding the physiological bases of fMRI and fNIRS techniques, with a comprehensive overview of how the techniques can be combined to provide an enhanced understanding of brain function in healthy controls and patient populations. Taken together, fMRI and fNIRS complement each other well, particularly for investigating neurovascular coupling and the neural correlates of cognitive functions among patient populations. Based on the findings outlined in this review, this multimodal approach may, for instance, aid in minimizing false-negative findings in BOLD fMRI in populations where CBF may be atypical, and allow for a better understanding of how the hemodynamic factors underlying the BOLD response might differ according to population variables (such as fitness level). As multimodal imaging gains popularity, increasingly sophisticated methods of analysis will likely aid the interpretation of fMRI-fNIRS data, further enabling the investigation of hemodynamic data, either concurrent or combined, in ways that would not be possible from either technique alone (Yuan and Ye, 2013). Although several groups have begun work in this area (Okamoto et al., 2004b; Moriai-Izawa et al., 2012), as some of the technical and practical considerations with fNIRS are better addressed, there may be less of a focus on “validation studies” and more investigations illuminating differences between cortical activation associated with everyday cognitive functions and fMRI-adapted paradigms.

## Author Contributions

VS primarily wrote and edited the manuscript. CB contributed to outline and written manuscript. CM heavily edited the manuscript and contributed new ideas. JRG contributed to the idea and outline of the manuscript as well as the written document.

## Conflict of Interest Statement

The authors declare that the research was conducted in the absence of any commercial or financial relationships that could be construed as a potential conflict of interest.
